# Parallel Odor Processing by Two Anatomically Distinct Olfactory Bulb Target Structures

**DOI:** 10.1371/journal.pone.0034926

**Published:** 2012-04-04

**Authors:** Colleen A. Payton, Donald A. Wilson, Daniel W. Wesson

**Affiliations:** 1 Department of Neurosciences, Case Western Reserve University School of Medicine, Cleveland, Ohio, United States of America; 2 Emotional Brain Institute, Nathan S. Kline Institute for Psychiatric Research, Orangeburg, New York, United States of America; 3 Department of Child and Adolescent Psychiatry, New York University School of Medicine, New York, New York, United States of America; Université Lyon, France

## Abstract

The olfactory cortex encompasses several anatomically distinct regions each hypothesized to provide differential representation and processing of specific odors. Studies exploring whether or not the diversity of olfactory bulb input to olfactory cortices has functional meaning, however, are lacking. Here we tested whether two anatomically major olfactory cortical structures, the olfactory tubercle (OT) and piriform cortex (PCX), differ in their neural representation and processing dynamics of a small set of diverse odors by performing *in vivo* extracellular recordings from the OT and PCX of anesthetized mice. We found a wealth of similarities between structures, including odor-evoked response magnitudes, breadth of odor tuning, and odor-evoked firing latencies. In contrast, only few differences between structures were found, including spontaneous activity rates and odor signal-to-noise ratios. These results suggest that despite major anatomical differences in innervation by olfactory bulb mitral/tufted cells, the basic features of odor representation and processing, at least within this limited odor set, are similar within the OT and PCX. We predict that the olfactory code follows a distributed processing stream in transmitting behaviorally and perceptually-relevant information from low-level stations.

## Introduction

Sensory perception requires that a transduced or internalized environmental signal be transmitted through a hierarchal information processing network [1], [2]. At some levels, sensory networks employ inter-regional anatomical divergence to distribute information. These distributed processing schemes can be highly advantageous. In the auditory system, for example, binaural information stemming from the auditory nerves, can be used as an index of input time differences or input level differences depending upon the pathway of input once departing the auditory nerve [3], [4]. In this case, post-synaptic recruitment of the medial superior olive is critical for interaural time differences and that of the lateral superior olive for interaural level differences. Uncovering whether similar anatomical divergences provide functional advantages to sensory systems will allow advances in our understanding of perception.

The mammalian olfactory system is rich with both hierarchal and distributed processing nodes [5]–[7]. Odorants are transduced within the nasal epithelium by olfactory receptor neurons [8], each expressing a single type of olfactory receptor [9]. Olfactory receptor neurons discretely converge onto glomeruli in the olfactory bulb (OB) at a ratio of ∼10,000∶1 [6]. From there post-synaptic mitral or tufted cells, each receiving information from a single glomerulus, project across great distances into higher-order structures, including the piriform cortex (PCX), olfactory tubercle (OT), anterior olfactory nucleus, and amygdala [10]–[15]. Recently, significant attention has been paid to the anatomical connectivity of OB mitral/tufted cells into higher-order olfactory centers [16]–[20]. These studies have highlighted that the seemingly ordered lay-out of connectivity and convergence within the OB is lost at the level of the cortex. Indeed, even output from a single glomerulus, or that of a single mitral/tufted cell, is dispersed, albeit uniquely, across numerous olfactory cortical structures [18], [19].

This differential projection of OB mitral/tufted cell afferents into down-stream structures provides an anatomical framework for the potentially unique representation and processing of odors within these structures. However, a direct test of this, or even a basic comparison of odor-evoked activity between olfactory cortices in rodents is lacking. Therefore, here we explored the spontaneous and odor-evoked activity in two olfactory cortices, the OT and PCX. We employed a relatively small but diverse array of odors at a single concentration each and addressed what aspects of odor representation and processing were divergent between these two structures. Contrary to our predictions, we found that single units in the OT and PCX represent odors in highly similar manners, including response magnitudes, breadth of tuning, and temporal dynamics. Taken in the context of this diverse but limited odor set, these data support the prediction that the olfactory code follows a distributed processing stream in transmitting behaviorally and perceptually-relevant information from low-level stations.

## Materials and Methods

### Experimental subjects

Adult male (*n* = 8) and female (*n* = 9) c57/BL6 mice (2–6 months of age) bred and maintained within the Nathan S. Kline Institute for Psychiatric Research animal facility were used. Food and water were available *ad libitum*. Mice were housed on a 12∶12 (light∶dark) cycle with all experiments performed during the light phase. All experiments were conducted in accordance with the guidelines of the National Institutes of Health and were approved by the Nathan S. Kline Institute's Institutional Animal Care Committee.

### *In vivo* electrophysiology

Mice were anesthetized with urethane anesthesia (1.0 mg/kg, I.P.) and supplied with atropine hydrochloride (25 mg/kg, I.M.) to minimize tracheal congestion. Mice were then mounted in a stereotaxic frame outfitted with a water-filled heating pad (38°C), the skin overlying the skull administered local anesthetic (1% xylocaine, S.C.) and later removed exposing the dorsal skull. A single craniotomy (∼3–4 mm squared) was performed over the OT and anterior-aspect of the PCX. A tungsten recording electrode (0.01″ O.D.; A-M Systems, Washington) was lowered into the PCX or OT at the start of the session and then later, raised and lowered into the alternate region. In this manner, the order of recording location was counterbalanced and this was performed in a pseudo-random order across all mice. Additionally, male and female mice were used in a semi-counterbalanced order throughout the study, with ∼1/2 of the males recorded before, and the other 1/2 recorded after female recordings.

**Figure 1:**
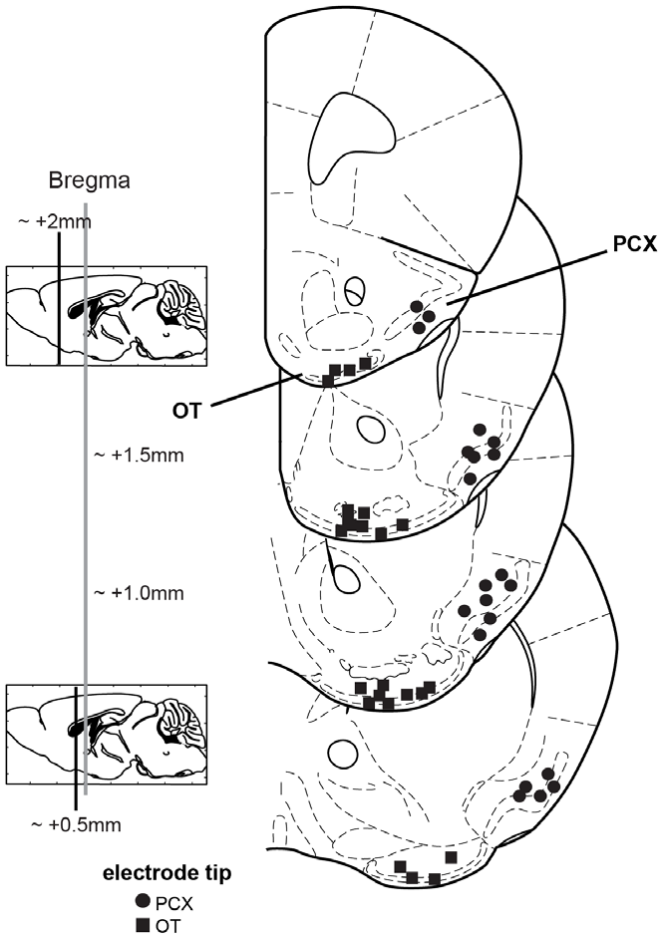
Electrode tip locations verifying extent of OT and PCX recording sites. Coronal stereotaxic panels showing the approximate location of electrode tips from records used for analysis. Coronal sections span from 2.0 – 0.5 mm anterior of bregma, in 0.5 mm intervals. Panels adapted from [61].

Following recordings, mice were transcardially perfused with 10% formalin and brains were removed and stored in 30% sucrose in formalin. Electrode locations were verified with post-mortem histological inspection of 40 µm coronal sections. Only recordings in which the electrode tips were found within the OT (layers i, ii, or iii) or PCX (layers ii or iii) were included for analysis in this study. As shown in Figure 1, OT recording sites (*n* = 24) were found mostly within the mesial aspect of the OT, spanning almost the entire anterior-posterior axis. In the PCX, recording sites (*n* = 21) were all found within anterior PCX (within sections containing the lateral olfactory tract). Recording electrode potentials along with stimulus presentation events were acquired using Spike2 software (Cambridge Electronic Design Ltd., Cambridge, England).

### Stimulus presentation

Odors were presented to anesthetized mice using an air-dilution olfactometer at 1 L/min flow using medical grade nitrogen. Stimuli included fox urine (www.predatorpee.com), freshly crushed mouse chow (Purina), male mouse urine, female mouse urine (see details below), and isoamyl acetate (Sigma Aldrich, St. Louis, MO). All liquid odorants were diluted 1∶10 in their liquid state, except isoamyl acetate which was undiluted. Odor∶Dilution flow proportion was 1∶10. Odors were presented in a counterbalanced manner 2 sec each, at a minimal 30 sec inter-stimulus interval (ISI) and were triggered off of the animal's respiration using a piezo foil placed under the animal's chest and a window discriminator to detect peaks of respiration (World Precision Instruments, Sarasota, FL). Individual stimuli were present for ≥5 trials.

Urine was collected and handled following previously established methods [21]. C57/BL6 mice (*n* = 10/sex, 2–4 mo of age) were held by the nape of their neck and urine samples collected into sterile odorless plastic tubes. Urine collected each day was pooled across all mice within each sex and frozen at −80°C. To minimize possible daily variations in urinary composition, daily urine samples were thawed and homogenized together. Homogenized samples were then stored in 100 ul aliquots at −80°C until experimentation.

### Data analysis

Electrophysiological data were analyzed as previously described [22], [23]. Single-unit spike sorting, cluster cutting and waveform analysis were all performed in Spike2 software (Cambridge Electronic Design, Ltd.). Verification of single-units was accomplished with a conservative inter-spike-interval threshold. No more than 1% of spikes from a single-unit could occur with an inter-spike-interval of less than 2 msec. Putative units which did not pass these criteria were omitted from further analysis.

Firing data both pre-odor (2 sec immediately prior to odor onset) and during odor (2 sec of odor on) were used for all analyses. These data across trials were often sorted within odors and reported in raw firing rate (Hz) or in normalized odor-evoked magnitudes (firing rate during odor as a proportion of that pre-odor). For the purposes of this paper, we defined odor signal∶noise ratios conservatively as the averaged odor-evoked spike magnitude divided by the standard deviation of the spontaneous firing. We also analyzed onset latencies of firing (tonset). This was calculated by measuring the time of onset of the first inhalation in the presence of odor to the time of the first action potential. All statistical analyses were performed in MATLAB (The MathWorks Inc., Natick, MA). Values are reported as mean ± standard error of the mean (SEM) unless otherwise stated.

## Results

### Spontaneous multi-unit activity in the OT and PCX

In the present study, we sought to test whether the OT and PCX, two anatomically prominent olfactory cortices, are unique in their representation and processing of odors. We recorded from a total of 63 isolated single-units in urethane anesthetized mice (Fig. 1; see Materials & Methods). Recordings were performed while the subjects were under anesthesia to reduce the likelihood of state-dependent modulation of unit firing (e.g., [24], [25]), which may not be equally dispersed throughout the cortex [26]. We recorded from a total of 32 OT (13 mice [15 male units, 17 female units]) and 31 PCX units (11 mice [16 male units, 15 female units]). Among these, 30 OT (94%) and 25 PCX units (81%) were spontaneously active (≥1 spike within 2 sec preceding any odor).

**Figure 2:**
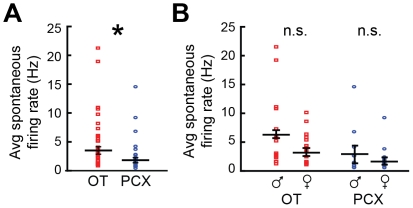
Spontaneous multi-unit activity in the OT and PCX. (**A**) Average spontaneous firing rates of OT (left) and PCX units (right). Each point represents average within units of 2 sec prior to odor, across 4–6 trials (*n* = 30 OT units, 25 PCX units). Horizontal bars = average ± SEM. *p<0.05, 2-tailed *t*-test. (**B**) Average spontaneous firing rates of males and female units within the OT (left) and PCX units (right). Same data as in (A). Horizontal bars = firing rate average across units ± SEM. n.s. = *p*>0.05, 2-tailed *t*-test.

We began by exploring whether OT and PCX units differed in their average spontaneous firing rate. OT units on average indeed displayed greater spontaneous firing rates than those in the PCX (*F*(1, 53) = 4.902, *p* = 0.03) (Fig. 2A). Recording from both males and females provided the opportunity to analyze for sex differences; however, while there was a trend for units in males to show higher spontaneous activity than females in both structures, this effect was not significant (*F*(1, 53) = 3.867, *p* = 0.05) (Fig. 2B). Thus, spontaneous multi-unit activity differs regionally within the olfactory cortex.

### Regionally-similar responsivity to odors in OT and PCX

**Figure 3:**
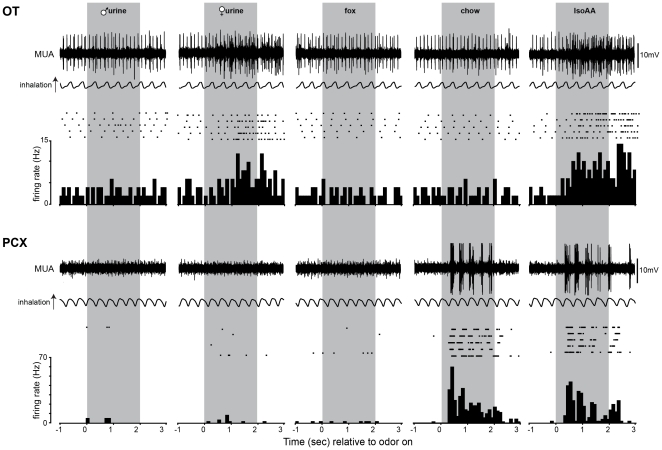
Example OT and PCX odor-evoked spike trains and stimulus histograms. Multiunit activity (MUA) from a single OT (top) and PCX (bottom) recording. Also shown in raster and peristimulus time histogram (PSTH) form is the activity of a single isolated unit (1 unit/region) across each trial of odor presentation. For these purposes, we selected a spontaneously active OT example which is representative of the greater spontaneous firing among OT units versus those found in the PCX (Fig. 2). In this example, the PCX unit burst more phasic with respiration, though this is not consistent across all units. Units from both structures in these examples responded to 2 of the 5 odors.

The PCX and OT both receive monosynaptic input from the OB [12], [14], [27]. Recent detailed anatomy displaying differential input of the OB into OT and PCX [18]–[20], and potentially different short-term dynamics of OB synaptic input to these structures [28], provides a foundation for possibly unique representations (responsivity, ensemble recruitment, etc.) of odors within these structures. We examined whether or not the OT and PCX differ in their odor responsivity by exploring unit firing upon presentation with a small array of odors (see Fig. 3). We focused on natural, ‘ethologically-relevant’ odors with the logic that a battery of these odors (mouse chow, mouse urine, predator urine) spans common processing spectrum of both of these structures. Each of the 4 ethologically-relevant odors (mouse chow, female mouse urine, male mouse urine, fox urine) and 1 less ethologically-relevant odorant (isoamyl acetate) were presented in a counter-balanced order >4 trials each to assess the responsivity of each unit to odors (see Materials & Methods).

**Figure 4:**
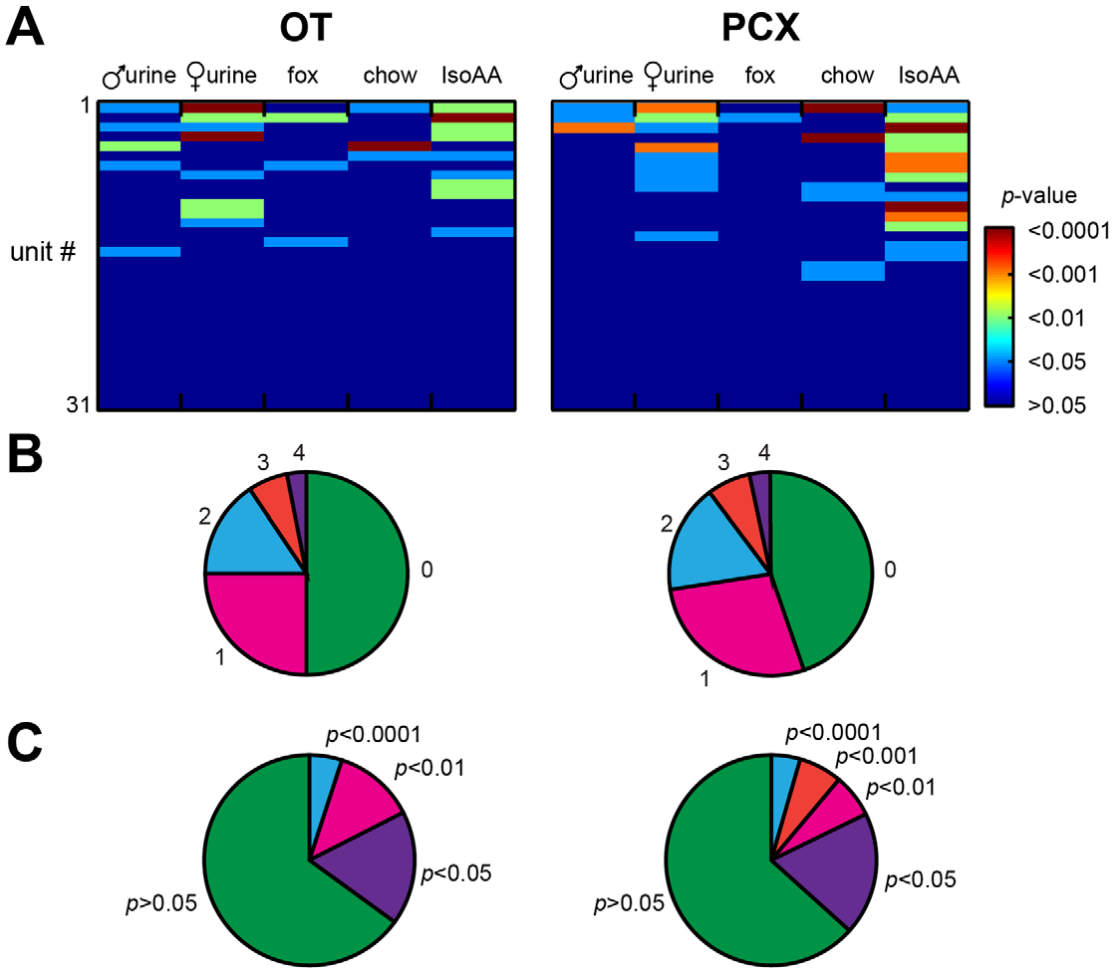
Odor-evoked response probability in the OT and PCX. (**A**) 3-dimensional histogram of odor selectivity among OT (left) and PCX units (right) in response to the five odorants (male mouse urine, female mouse urine, fox urine, chow, IsoAA; see Materials and Methods). *p* value = 2-tailed *t*-test of spiking 2 sec pre-odor vs. 2 sec during odor (≥4 trials/odor/unit). Units arrangement (#1,2,3, etc.) is based upon average magnitude of significance across odors. Whereas some odors evoked significant responses throughout numerous units (e.g., IsoAA), others (e.g., fox urine) did not. (**B**) Pie charts of unit tuning in OT and PCX. 0 = not responsive to any odor, 4 = responsive to 4 odors (no unit responded to all 5 odors). 50% of OT and 58% of PCX units displayed a significant modulation in firing rate to at least one odor. Same data as in (A). (**C**) Pie charts of response magnitudes of odor-evoked activity in OT and PCX. Same data as in (A).

As shown in Figure 4A, units responsive to each odor were found within both the OT and PCX. To begin quantifying odor-evoked responses, first we analyzed the degree of modulation in firing rate among cortical units. 50% of OT (16 of 32) and 58% of PCX (18 of 31) units displayed a significant modulation in firing rate in response to at least one of the odors (*p*<0.05 spike increase or decrease within 2 sec during odor compared to 2 sec pre-odor) (Figs. 4A and B). Out of these, only one unit displayed significant suppression in response to odor, with the remaining units being excitatory. Units within both regions possessed similar tendencies to display either selective (responsive to only 1 odor) or broad, indiscriminate odor tuning (Figs. 4A & B). Additionally, across all odors, the magnitude of evoked activity (indexed by *p*-values) was similar between OT and PCX (Fig. 4C). Thus, the OT and PCX represent odors (at least those used in this limited array) using shared principles of odor responsivity including breadth of tuning and odor-response magnitudes.

Despite similar manners of odor responsivity, the OT and PCX may uniquely represent particular odors or possibly the intensity of each odor. For instance, 45% of PCX units were responsive to IsoAA, whereas only 28% of OT units were (Fig. 4A). Further, 19% of PCX units were responsive to mouse chow, but only 9% of those in OT were (Fig. 4A). However, across all odors, OT and PCX units displayed similar probabilities of responding (Χ^2^ (4, *n* = 315) = 3.82, *p*>0.05). Thus, in agreement with predictions based upon anatomical tracing studies [18]–[20], at the individual odor level, olfactory cortical structures may each display unique patterns of odor representations.

**Figure 5:**
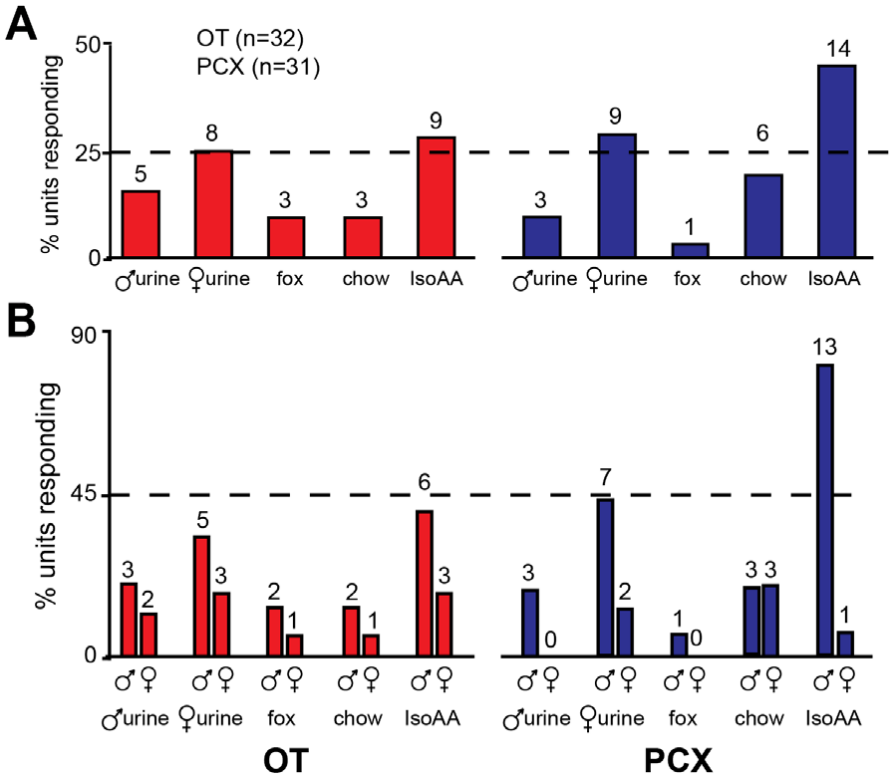
OT and PCX units show odor-specific and sexually-differentiated odor responsivity. Histogram of OT and PCX odor response data calculated in ‘% responding’ (as in Fig. 4A), with male and female subjects grouped (**A**) and sorted by sex (**B**). Numbers above bars = # units responding.

We also took advantage of male and female subjects and explored whether there were sex differences among the OT and PCX units in their odor representations. Across both structures, male units were more responsive to odors in comparison to those of females (Χ^2^ (4, *n* = 315) = 14.87, *p*<0.05) (Fig. 5). This sex difference was more pronounced for certain odors. For example, a striking 81% of male units in the PCX were responsive to IsoAA, whereas only 6% of female units were. Further, 44% of male units in the PCX were responsive to female urine, whereas only 11% of female units were. While similar sex differences were observed in the OT, the most profound examples were observed in the PCX (Fig. 5). Thus, cortical responses to odors, which are unique within each cortex, are further diversified by sex.

### Odor-evoked firing rate and response magnitudes in OT and PCX

**Figure 6:**
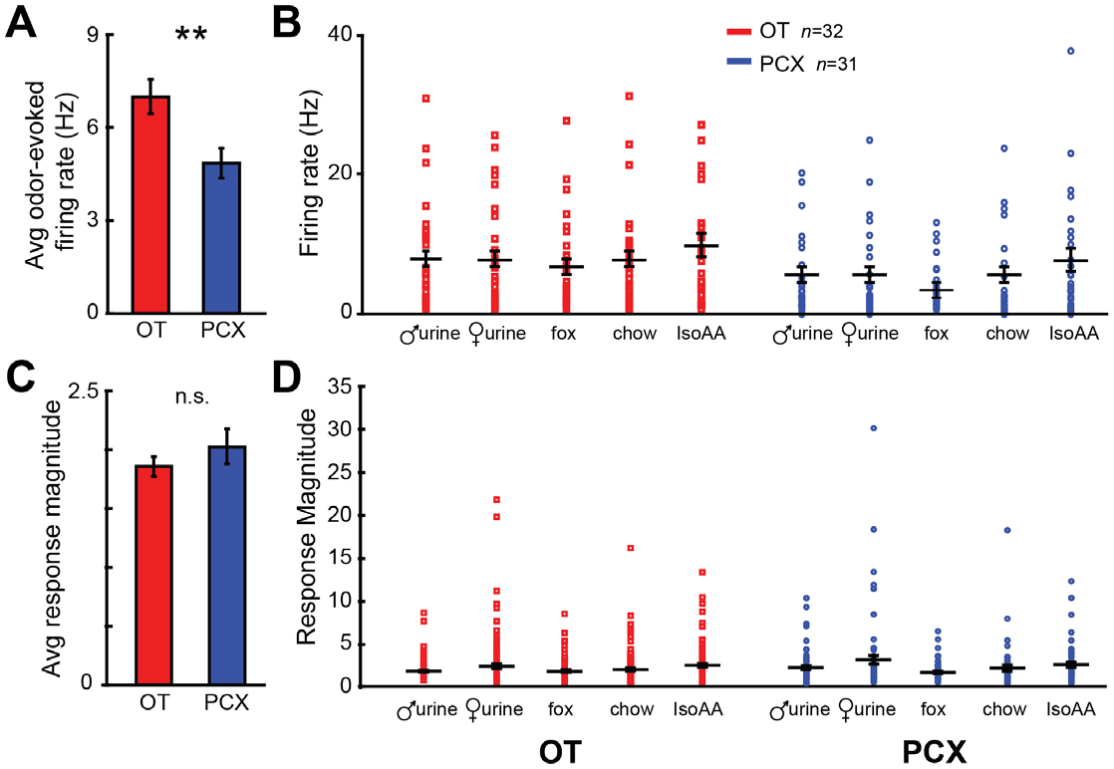
Odor-evoked spike rate, but not response magnitudes are greater in OT than PCX. (**A**) Average odor-evoked firing rate of OT (left) and PCX units (right). Data ± SEM. **p<0.01, 2-tailed *t*-test. (**B**) Same rate data as in (A) but organized by odor. Each point represents average of 2 sec during odor, across >4 trials/unit. Horizontal bars = firing rate average across units ± SEM. (**C**) Average odor-evoked response magnitude (firing rate during odor as a proportion of that pre-odor) of OT and PCX units. Data ± SEM. n.s. = *p*>0.05, 2-tailed *t*-test. (**D**) Same magnitude data as in (C) but organized by odor. Each point represents average of 2 sec prior to odor, across >4 trials/unit. Horizontal bars = average ± SEM.

Regional comparisons of odor-evoked firing activity and response magnitude in the OT and PCX may provide insights into manners of odor coding within these structures. Therefore, we explored whether the OT and PCX differ in their odor-evoked firing rates and response magnitudes. First, we analyzed the average odor-evoked firing rate among OT and PCX units. As shown in Figure 4A, there was a regional difference in the average odor-evoked firing rate across all odors, with the OT having a greater firing rate than PCX (*F*(1, 313) = 8.413, *p* = 0.004). This tendency for OT firing rate to be greater than PCX was generally conserved across all odors (Fig. 6B), perhaps attributable to the heightened spontaneous firing rate observed among OT units (Fig. 2). Indeed, after normalizing for the differences in spontaneous firing rates by converting the odor-evoked data into odor-evoked response magnitudes (Fig. 6, see Materials and Methods), OT and PCX responses, across all odors, did not differ (*F*(1, 1024) = 1.215, *p* = 0.27) (Fig. 6C). These data demonstrate that the gross, but not normalized amounts of odor-evoked activity differ between OT and PCX.

### OT and PCX odor signal∶noise

**Figure 7:**
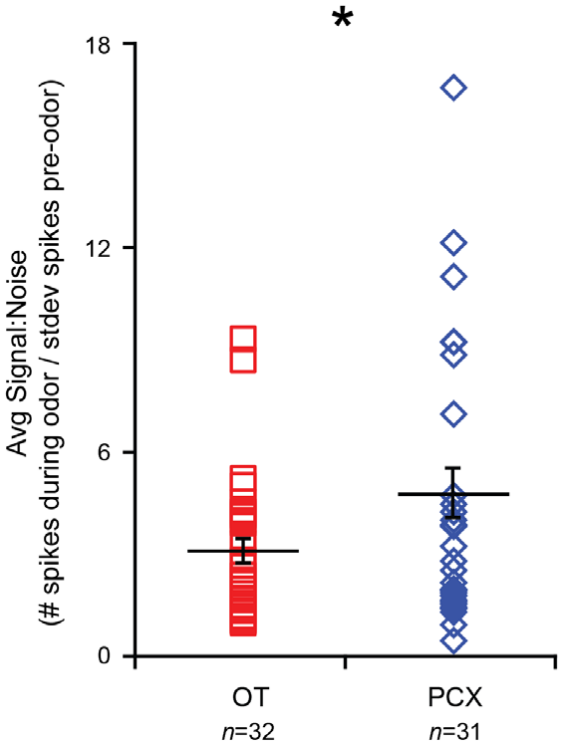
OT and PCX Odor signal∶noise. (**A**) Average signal∶noise (s∶n, see Methods) in OT and PCX units. Each point represents average s∶n of each unit, across all odor presentations (>4 trials/unit) and odors. Horizontal bars = average s∶n across units ± SEM. *p<0.05, ANOVA followed by Fisher's PLSD.

The enhanced spontaneous activity, yet lesser odor-evoked response magnitudes in the OT compared to PCX (e.g., Figs. 2 & 6) suggests that there is a differential signal∶noise property in the OT which is not present in PCX. A determination of odor signal∶noise differences between these structures may elucidate unique manners wherein these structures contribute to odor quality perception (e.g., [29]–[32]). Therefore, we calculated the mean odor-evoked rate as a proportion of the standard deviation of the spontaneous firing rate for each unit (see Materials & Methods). We found that PCX units responded to odors with a higher signal∶noise than those in the OT (*F*(1, 60) = 4.587, *p* = 0.036) (Fig. 7). This difference was mostly attributable to about 25% of PCX units which showed signal∶noise ratios greater than any found within the OT (>4–16.5). Thus, one difference between OT and PCX is found among the strength of odor input relative to tonic firing.

### Dynamics of odor-evoked activity in OT and PCX neural ensembles

**Figure 8:**
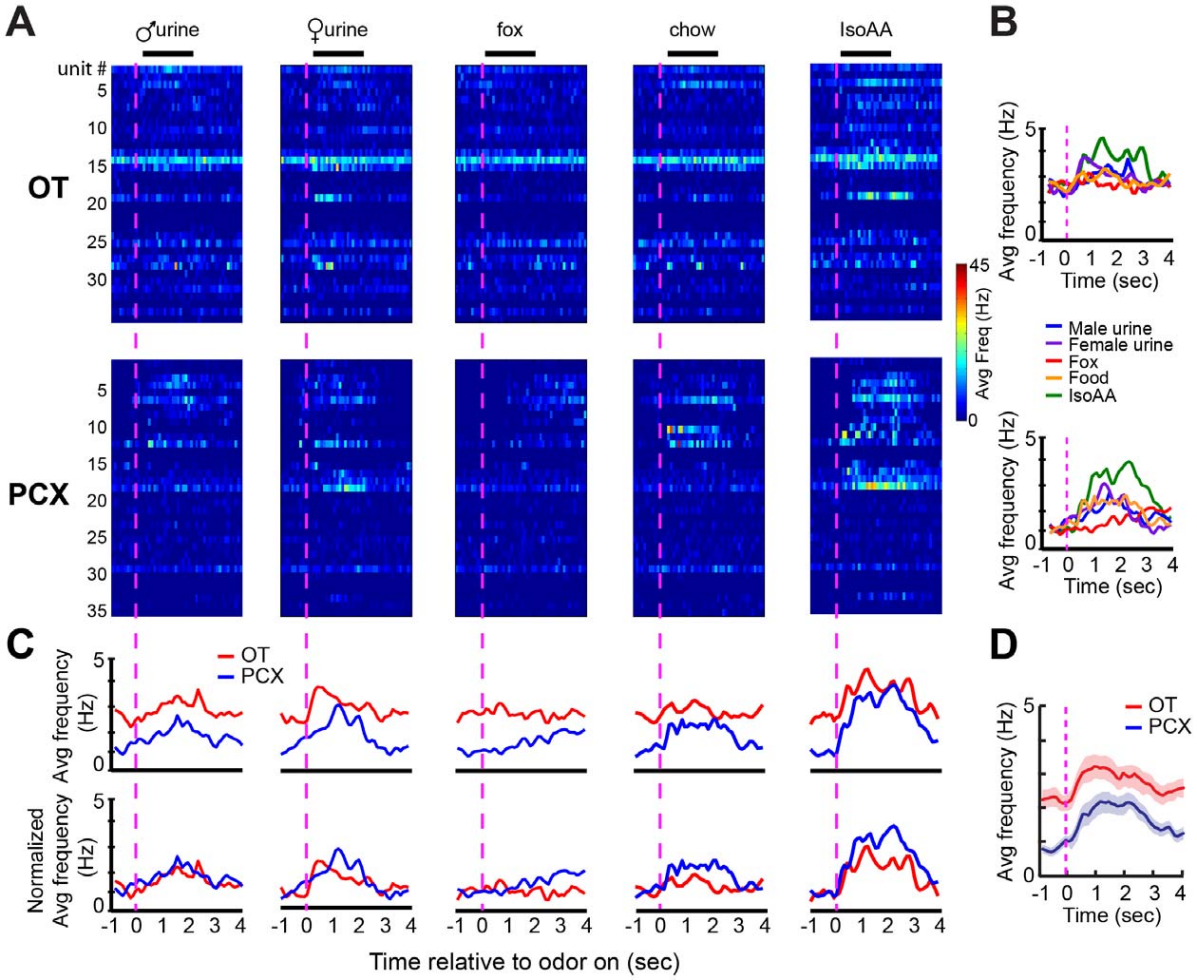
Temporal dynamics of odor-evoked activity in OT and PCX neural ensembles. (**A**) 3-dimensional histograms of average firing frequency for each unit across the five odors within the OT (top) and the PCX (bottom). Diversity of intensity in spontaneous firing and odor-evoked changes in firing are visible within both structures. Units arrangement (#1,2,3, etc.) is arbitrary but consistent across panels within regions. (**B**) Average firing frequency of OT (top) and PCX (bottom) units relative to odor onset sorted by odors. (**C**) Raw (top) and normalized (bottom) average firing frequency of OT and PCX units sorted by odors (left to right). (**D**) Average firing frequency across all odors and units. Data displayed as mean ± SEM. Vertical magenta dashed line = odor onset.

The time-course of sensory neuron activity is hypothesized to play a major role in shaping perception and decisions (e.g., [33], [34]). This is especially the case in the olfactory system wherein odor information is constrained from entering brain by the necessary inhalation of an odor, after which odor identification occurs rapidly [35], [36]. Due to this, we next examined whether or not there were regional differences in the temporal nature of odor-evoked activity between the OT and PCX. Time differences in odor responses in either of these regions would provide a neural substrate a unique locus for rapid reactions to odors. Similar to that addressed within previous figures (Figs. 3 & 4), each odor evoked unique activity, differentially within the OT and PCX, in a unit-dependent manner. While instantaneous firing rate among individual OT and PCX units varied substantially (Fig. 8A), at the ensemble level, patterns of odor-evoked activity shared a generally common onset and offset function dependent upon the time-course of the odor (Figs. 8B & C). Following normalization to baseline firing rates, no differences between the OT and PCX in the distribution of odor-evoked responses throughout the odor presentation were detectable (Fig. 8C). Instead, units within both structures appeared to display a similar odor-evoked temporal response dynamic (Fig. 8D).

**Figure 9:**
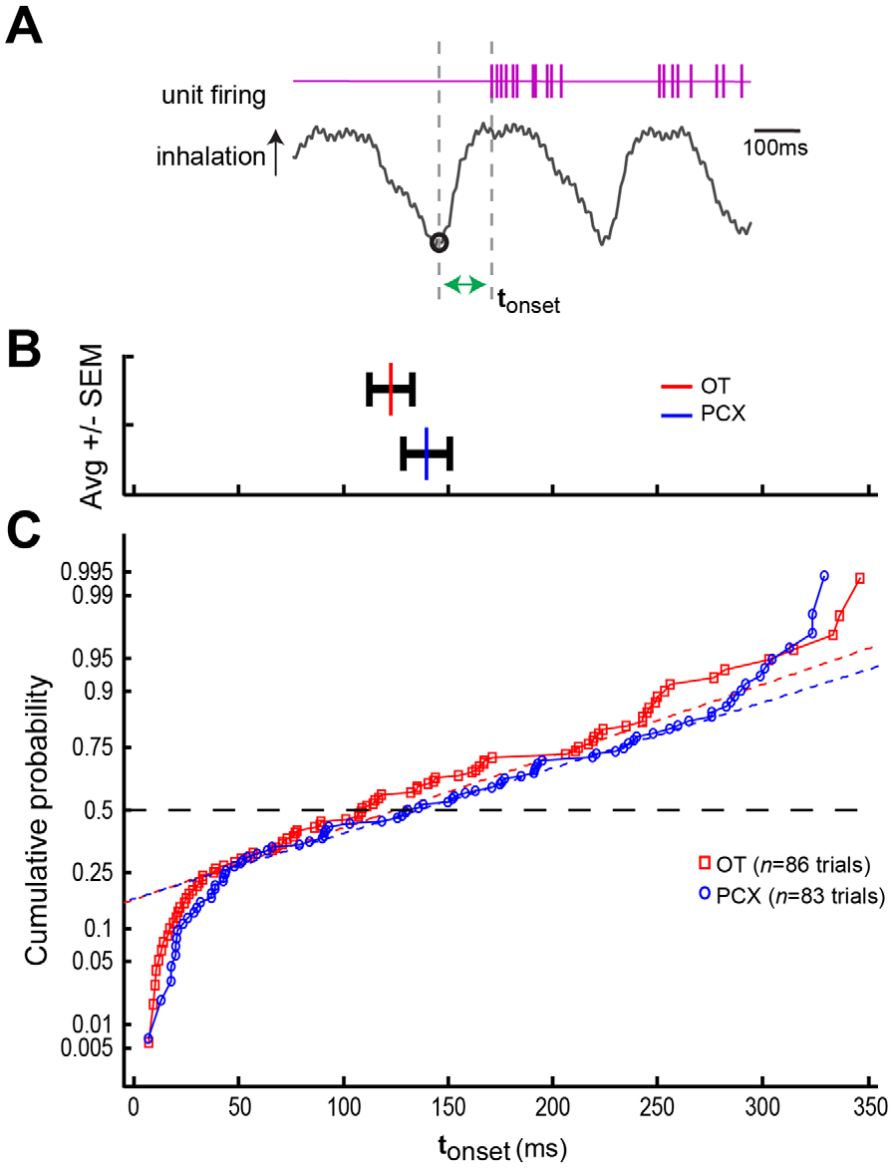
Onset of odor input is similar between OT and PCX. (**A**) Example traces of unit firing and respiration over the course of 1 sec. Inhalation in respiratory trace is an upward deflection. Hash marks represent unit responses (spikes). t_onset_ = latency from first inhalation initiation during odor (circle) until the time of the first action potential (depicted here by the green horizontal arrow). (**B**) Average t_onset_ values ± SEM within the OT (red) and PCX (blue). (**C**) Cumulative probability plot of t_onset_ values (same data as in (B)). Dashed lines = normal fits of OT (red) and PCX (blue) cumulative probability data.

To more specifically test whether the OT and PCX differ in their temporal representation of odors (as suggested in Fig. 8D), we analyzed the response latency for the onset of odor-evoked activity. Onset latency was measured from the time of onset of the first inhalation in the presence of odor to the time of the first action potential (see Materials and Methods) (Fig. 9A). Measurements were made for all odor-responsive units (*n* = 16 OT, 18 PCX) across all odors for which the unit responded with a significant excitation (*p*<0.05 compared to 2 sec pre-odor, same significant odor-cell pairs as displayed in Fig. 4A). With the average respiratory frequency ranging from approximately 2–3 Hz, we selected to exclude all onset latencies exceeding 350 ms from statistical analysis under the logic that these reflect later-responding multi-synaptic responses versus presumptive direct input from the OB, consistent with previous cortical unit onset reports [37]. Out of these, a subset of onset latencies was <50 ms, likely reflecting spontaneous action potential firing. The mean onset latency for OT units was 125.9±95.2 msec (*n* = 86 trials, 6 mice) and for PCX units 142.6±100.2 msec (*n* = 83 trials, 10 mice) (Fig. 9B). While OT onset latencies were slightly less, there were no significant differences between regions in onset latencies (*F*(1, 167) = 1.229, *p* = 0.27) (Fig. 9B) nor their distributions (*p*>0.05, two-sample Kolmogorov-Smirnov test) (Fig. 9C). Although limited within this small array of odors, sorting onset latencies by odor revealed that onset latencies were not significantly different depending upon odor. For instance, latency of IsoAA to evoke spiking was similar within OT and PCX (*F*(1, 56) = 0.339, *p* = 0.56). Similarly, no effect of sex was found on onset latencies across all odors to either the OT or PCX (*F*(1, 167) = 2.426, *p* = 0.12). Thus cortical odor input evolves more-or-less equally within the OT and PCX for about the first 200 msec following odor inhalation.

## Discussion

The majority of what we know about differences between olfactory cortical structures beyond anterior and posterior piriform cortex [29], [31], [38], stems from anatomical work [12]–[14], [18]–[20], [39]. In contrast, studies exploring whether or not the diversity of anatomical connections from the OB to olfactory cortices have functional meaning are rare (although see [28]). Addressing this gap, here we performed an investigation into possible differences in odor processing between two anatomically major olfactory cortical structures, the OT and PCX. The OT and PCX both receive monosynaptic input from OB mitral/tufted cells [13]. Whereas both cell types provide afferent input, mitral cells are the primary source of input into PCX [40], [41] with only the anteroventral aspect receiving input from tufted cells [13]. In contrast, the OT receives extensive input from OB tufted cells, with much less from mitral cells [27], [40], [42]. Thus, the basic foundations of odor input to these cortices differ (but see below). Several other notable differences intrinsic to these structures are also present. These include the lack of an association fiber network in the OT, and differing levels of critical neurotransmitters and patterns of neurotransmitter receptor expression (for review see [43]).

Perhaps reflecting these gross differences we found that the OT and PCX differed at several basic levels. First, units in the OT displayed an approximately 15% heightened spontaneous firing rate compared to those in the PCX. This likely was responsible for yielding significant differences between the OT and PCX in odor-evoked firing rates (Fig. 6A), since the spontaneous rate off-set the rate during odor. Indeed, normalized data did not differ between structures (Fig. 6B). Additionally, we found that the proportion of units in the OT responsive to certain odors differs from those in the PCX (Fig. 4). These differences were in most cases subtle within this small collection of units (∼30/structure), yet may have larger implications across the entire population of cortical cells. Finally, we found a sex difference in unit responsivity to some odors which was in at least one case two-times greater in the PCX than the OT (Fig. 5B). Sexually-differentiated odor-evoked unit responses among olfactory cortex neurons, as far as we know, are unreported. This finding complements other work demonstrating sexually-differentiated olfactory responses in the OB [44]. Here we found that the OT and PCX may be uniquely sexually-differentiated, as suggested by previous studies of sex hormone receptor localization [45], [46]. While in the present study we did not control for fluctuations in endogeneous hormone levels (our animals were gonadally intact), this finding provides a foundation for possibly sexually-differentiated pathways of odor processing onto higher-order structures (amygdala, habenula, entorhinal cortex) and thus also for previous reports of sex-specific odor-guided behaviors [21], [47]–[49].

The enhanced signal∶noise found within the PCX versus OT, and the minor yet significant differences in activity rates between these structures may be expected to impact how downstream sites respond to odor stimulation. The OT and PCX have different projection targets, with the OT targeting regions important for motivated behavior and reward such as the nucleus accumbens and lateral habenula [50]–[52], while the PCX mostly innervates areas important in memory, such as the entorhinal cortex [53], [54]. Though normalized sensory output of the OT and PCX are comparable, the relatively higher tonic and odor-evoked activity of OT units may shape activity in its targets differently than PCX output (as suggested by the greater PCX signal∶noise). Indeed, a possible disadvantage of large tufted cell input to the OT, and consequently enhanced detection capacity, is a sacrifice in dynamic range and possible odor quality coding within the OT as reported in the PCX [29]–[31], [55]. Additional differences between the OT and PCX may be obscured by the use of anesthesia, thus recordings in animals performing different behaviorally relevant tasks may be especially useful in dissecting the unique contributions of these structures. However, it should be noted that olfactory regions as anatomically distinct as the orbitofrontal cortex and the PCX show remarkably similar activity patterns in behaving rats [56]. Olfactory areas, including the OT and PCX, are highly interconnected and modulate each other's activity [57]. Complete understanding of the role of specific regions in odor coding may require reversible silencing of one region to see the activity of the other in isolation.

### A parallel distributed olfactory code

While multiple differences were detected between OT and PCX, they were surprisingly subtle given the major anatomical and neurochemical differences between these structures [43]. Indeed, at least in the context of the odor screen used here, the OT and PCX shared similar breadths of odor responsivity (units in OT were equally capable of discriminating between odors as those in PCX), and similar latencies to odor responses. Thus, the basic aspects of odor processing in the OT and PCX do indeed appear to operate along common principles and support the prediction that, after leaving the OB, the olfactory code follows a distributed processing stream in transmitting behaviorally and perceptually-relevant information from low-level stations. This finding is reminiscent of those reported in invertebrates [58] and may be critical for adaptive behaviors in dynamic odor environments. It is also interesting to consider, that despite the major anatomical differences in OB input outlined above, subsets of OB neurons have been discovered to innervate both the OT and PCX [17], [19] – thereby providing a possible substrate for the similar distributions of odor responsivity we found within these structures.

Here we provided a preliminary investigation into physiological differences between OT and PCX units by assessing responsivity to a limited, though diverse odor array. Several outstanding questions remain however. For instance, do the OT and PCX differ in their representation of odor concentration? OT units might be predicted to have very low thresholds given the large convergence of low-threshold tufted cells into the OT [43], [59]. In fact, the restricted use of relatively high concentration odors here may have reduced the differences between the structures which may be more apparent nearer threshold. Further, our experiments employed only a small array of odors, and did not address differences in fine odor discrimination, nor experiential-induced changes in odor discrimination. Whether the OT plays an active role in odor discrimination at the behavioral level is unknown, yet it would be interesting to test given its lack of association fiber network [60]. Also, the possibility that OT and PCX units differentially fire with the phase of respiration (as perhaps observed in Fig. 3), or along different aspects of the respiratory phase remains to be explored. Finally, as noted above whether the OT and PCX operate under different principles of odor processing in the awake-behaving state remains to be explored. Indeed, the similarities and relatively minor dissimilarities in odor processing features between the OT and PCX call into question whether or not the true differences in the roles of these structures for olfaction may be manifest only in the context of behavioral demands, wherein cortical activity may require multiplexing behavioral variables with odor responses.
