# Correction: Parallel Odor Processing by Two Anatomically Distinct Olfactory Bulb Target Structures

**DOI:** 10.1371/annotation/eb15723f-2df7-4cd6-8113-c565652d0628

**Published:** 2012-08-06

**Authors:** Colleen A. Payton, Donald A. Wilson, Daniel W. Wesson

Rampin et al. recently published a report also describing social odor evoked activity in the olfactory tubercle:

Rampin O, Bellier C, Maurin Y. Eur J Neurosci. 202 Jan;35(1):97-105.

We regret not citing the publication by Rampin et al. Although the Payton et al. and Rampin et al. articles utilized different techniques and stimulus sets, together they begin to establish olfactory sensory coding by olfactory tubercle neurons. 

